# The Buffalo Providence Insane Asylum

**Published:** 1861-09

**Authors:** 


### THE BUFFALO PROVIDENCE INSANE ASYLUM.

We take great pleasure in announcing to the profession the opening of this Institution, under the supervision of the SISTERS OF CHARITY. The crying necessity for such an institution has long been felt, not only by physicians who have been obliged to send patients long distances, but more fully still by the friends of this unfortunate class of patients, who have been obliged to incur the expense and trouble of removing their relatives far from them, until they could recover from a disease which cannot be properly treated outside the walls of an institution constructed with particular reference to the care and treatment of mental diseases.

This asylum is very pleasantly situated on Main street, near the termination of the railroad, making it convenient, and at the same time affording the retirement so desirable in the care and proper management of the Insane. The rooms are pleasant, attractive, well ventilated apartments, safe, and yet presenting no appearances indicating confinement or restraint.

The building and grounds when fully completed, will constitute one of the most pleasantly arranged Asylums in our country, in every feature desirable and attractive.

It is under the immediate superintendence of Sister ROSALINE BROWN, to whose energy, capacity and perseverance we are largely indebted for this charity. This Institution would seem to be denominational in character, but we wish to assure our medical friends, and through them all others who may be interested, that it is designed to be in no way exclusive or sectarian in any of its arrangements or benefits, but is open to all who may require its advantages, without any distinctions whatever, and will offer in an eminent degree all the opportunities which can possibly be afforded in any Insane Asylum.

The proper medical attendance of the insane constitutes one of the most important inducements which usually influence physicians to recommend patients to the care of those, who, connected with Insane Asylums, have extensive opportunity of observation, and who make this branch of medical practice, a subject of especial study. We congratulate the friends of this Asylum in obtaining the services of Prof. JAMES P. WHITE, whose unsurpassed ability will leave no doubts in the minds of *any*, as to their friends receiving the very best possible advice and medical care. Prof. WHITE'S reputation with the profession, will constitute an important attraction to this institution, forming an additional reason for physicians having patients requiring such retreat, to recommend and favor the BUFFALO PROVIDENCE INSANE ASYLUM.

